# UV and Visible Light-Induced Photocatalytic Efficiency of Polyaniline/Titanium Dioxide Heterostructures

**DOI:** 10.3390/molecules30010023

**Published:** 2024-12-25

**Authors:** Yongqiang Fu, Marcin Janczarek

**Affiliations:** Institute of Chemical Technology and Engineering, Faculty of Chemical Technology, Poznan University of Technology, Berdychowo 4, 60-965 Poznan, Poland; yongqiang.fu@doctorate.put.poznan.pl

**Keywords:** photocatalysis, titanium dioxide, polyaniline, conducting polymers

## Abstract

The concept of using polyaniline/titanium dioxide heterostructures as efficient photocatalysts is based on the synergistic effect of conducting polymer and metal oxide semiconductors. Due to inconclusive literature reports, the effect of different polyaniline/TiO_2_ ratios on photocatalytic activity under UV and visible light was investigated. In most papers, non-recommended dyes are used as model compounds to evaluate visible light activity. Therefore, colorless phenol was used instead of dyes in this study to clarify the real visible light-induced photocatalytic activity of polyaniline/TiO_2_ composites. This publication also includes a discussion of whether materials derived from bulk (non-nanostructured) polyaniline and TiO_2_ by the standard in situ oxidative polymerization method are suitable candidates for promising photocatalytic materials. The evaluation of photocatalytic activity was performed in both UV and visible light systems. X-ray diffraction and UV-Vis diffuse reflectance spectroscopy methods were applied to characterize the obtained samples. Obtained polyaniline (pure and in composites) was identified as emeraldine salt. In the UV system, none of the prepared samples with different polyaniline–titania ratios had activity better than reference P25 titania. It has been observed that the presence of polyaniline adversely affects the photocatalytic properties, as the polyaniline layer covering the titania surface can shield the UV light transmission by blocking the contact between the TiO_2_ surface and organic molecules. In the case of using visible light, no synergies have been observed between polyaniline and titania either. The photodegradation efficiencies of the most active samples were similar to those of pure polyaniline. In conclusion, in order to obtain efficient polyaniline/titania photocatalysts active in UV and/or visible light, it is necessary to take into account the morphological and surface properties of both components.

## 1. Introduction

Since the discovery of the Honda–Fujishima effect in the 1970s, research on titanium dioxide (TiO_2_; titania) has become a key topic in heterogeneous photocatalysis [1]. Another highlight of TiO_2_ research was its application for the remediation of environmental pollutants in 1977 when Frank and Bard reported photocatalytic oxidation of cyanide ions in water [2]. This has resulted in a steadily increasing number of publications on the use of titania as a photocatalyst in water and air environmental purification processes, meeting the continuing demand for this type of remediation technologies [3,4,5] and, furthermore, driven by photocatalysis synthesis of chemical compounds and renewable energy processes such as water splitting for hydrogen production confirm the wide range of titania possible applications [6,7,8]. The high interest in this material is due to its advantages, such as high photocatalytic activity, low cost, abundance, relative nontoxicity, and resistance to photocorrosion [9]. On the other hand, the weaknesses of TiO_2,_ such as the requirement of UV light to activate photocatalytic reactions (due to its wide bandgap) and the adverse phenomenon of charge carriers recombination, are factors limiting wider applications than expected, e.g., use only in regions with a high intensity of solar radiation [10,11]. Considering the above-mentioned limitations, there are various strategies for titania modification (surface modification, doping with metals or non-metals, self-doping, and heterojunction systems) [12,13,14,15,16,17,18,19,20,21].

Surface modification of TiO_2_ with organic sensitizers is one of the most promising directions for the activation of this semiconducting material toward visible light. An important example of this is the application of conducting polymers [22,23]. These compounds have a conjugated structure, which is a prerequisite for the formation of delocalized electronic states (π electron delocalization along the polymer backbone). This main property determines their unique electrical and optical properties [24,25]. Polyaniline (PANI) belongs to the conducting polymers group and has many advantages, such as low cost and high environmental stability. Furthermore, various structural forms of PANI can be distinguished, e.g., emeraldine base (EB) and emeraldine salt (ES). They differ in such important properties as conductivity and band gap energy [23]. ES can be classified as a p-type semiconductor. Its band gap (2.7 eV) is connected with the charge transfer exciton, such as the transition from the HOMO (π_b_) of the benzonoid rings to LUMO (π_q_) [23,26]. The design of PANI/TiO_2_ systems is based on the concept of coupling of n-type (TiO_2_) with p-type (PANI) semiconductors. The positions of bands of PANI and TiO_2_ determine the heterojunction type II [27]. Therefore, in combination with TiO_2_, PANI can improve the absorption of visible light and enhance the separation efficiency of photo-generated electron-hole pairs, thus boosting the photocatalytic performance under visible light [28,29].

However, due to the different properties of metal oxides and conductive polymers, integrating them into an effective nanocomposite is not straightforward. To solve this problem, it is necessary to implement intricate material design and preparation techniques to achieve a tight bond and synergistic effect between metal oxides and conductive polymers. There are research reports on PANI/TiO_2_ heterostructures as visible light-active materials that report these synergistic effects [30,31,32,33,34,35,36,37,38,39,40,41,42,43,44]. However, in most studies, organic dyes are the model compounds for visible light photocatalytic activity tests, which is not recommended and may distort the interpretation of final results [45]. Another issue is the overly diverse reports on the effect of different PANI/TiO_2_ ratios on the photocatalytic properties of the resultant heterostructures. Furthermore, the form of prepared PANI (e.g., emeraldine salt or base) is often not reported. This study also includes a discussion on whether composites derived from bulk (non-nanostructured) polyaniline and TiO_2_ are suitable candidates for promising photocatalytic materials using a standard in situ oxidative polymerization method.

To address the above-mentioned issues, the present study describes the influence of different molar ratios of PANI and titanium dioxide (commercially available P25) on photocatalytic activity both in UV and visible light systems. The standard in situ oxidative polymerization method was selected for the preparation of PANI/TiO_2_ heterostructures. Prepared samples were characterized by the X-ray diffraction method, mainly to confirm the form of PANI, and UV-Vis diffuse reflectance spectroscopy to describe the absorption properties. This comprehensive analysis helps to understand the impact of these preparation conditions on the performance of PANI/TiO_2_ composites to verify the possible presence of synergy effect between polyaniline and titania using a standard in situ oxidative polymerization method and bulk PANI and TiO_2_ input materials.

## 2. Results and Discussion

### 2.1. Characterization of PANI/TiO_2_ Heterostructures

**Figure 1:**
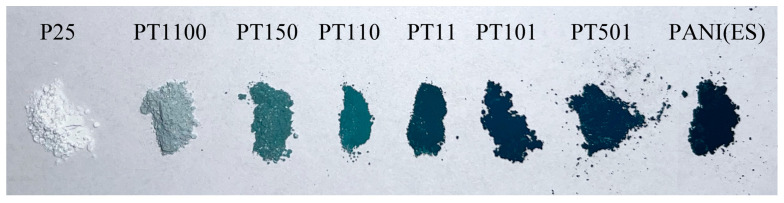
The color change of PANI/TiO_2_ samples.

Commercially available P25 titania was used as a base to prepare the resultant photocatalysts (PANI/TiO_2_) by in situ oxidative polymerization of aniline. Different molar ratios between aniline and P25 were investigated: 1:100, 1:50, 1:10, 1:1, 10:1, 50:1, and 100:1. The color of PANI-TiO_2_ composites varies depending on the oxidation state of PANI and the interaction with titania. PANI, in its leucoemeraldine (LEB) state, appears as white or pale yellow. In the pernigraniline (PB) state, the material is purple or deep blue. In the EB state, the undoped semi-oxidized form usually appears blue or bluish-purple. Considering the ES form of polyaniline, a green color was reported [46,47]. The bare PANI obtained by the oxidative polymerization method had a dark green color, which is in agreement with the literature reports on the ES form (Figure 1). With varying PANI:TiO_2_ ratios, the resultant composite colors changed gradually. At higher PANI contents (100:1, 50:1, and 10:1), the samples exhibited deep dark green shades, closely resembling pure ES. At a balanced ratio (1:1), the color turned to green, reflecting the mixture of PANI and TiO_2_. At lower PANI contents (1:10, 1:50, and 1:100), the samples became progressively lighter, appearing light green and grayish-green for PT150 and PT1100 samples, respectively. The color gradient highlights the diminishing visual contribution of PANI as its content decreases (Figure 1).

**Figure 2:**
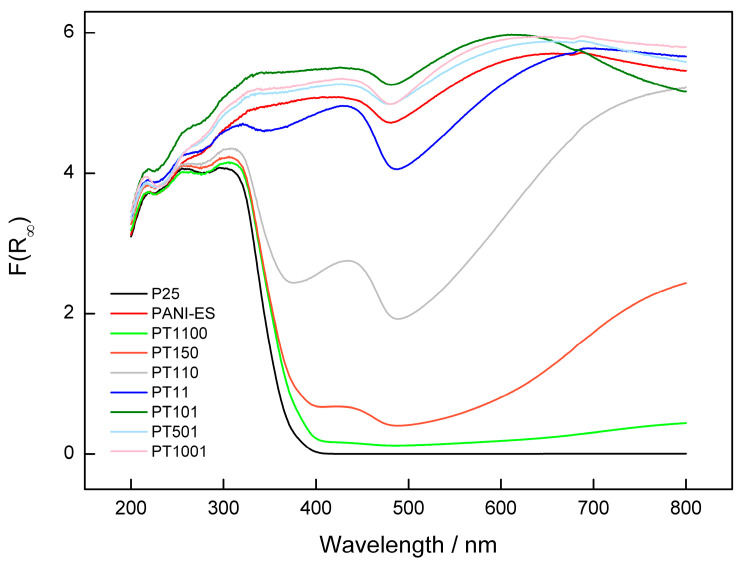
UV-Vis diffuse reflectance spectra of PANI/TiO_2_ samples.

UV-Vis diffuse reflectance spectra of obtained PANI/TiO_2_, pure PANI-ES, and P25 samples are shown in Figure 2. It is clearly visible that PANI/TiO_2_ materials absorbed both UV and visible light, whereas unmodified P25 absorbed only UV light. The DRS spectrum of PANI-ES was in agreement with the spectrum reported in the literature [48]. Two absorption peaks around 385 nm and 638 nm were visible on this spectrum. The first was related to the π→π* transition on the PANI chains, and the second one was contributed to polaron after proton doping and corresponds to the localization of electrons [49]. It is important to mention that for polyaniline–titania heterostructures with lower PANI:TiO_2_ ratio (PT150, PT110, and PT11), the peak at 385 nm red-shifts to ca. 430–440 nm, and the peak at 638 nm was replaced by a broad absorption band with a long tail. In the case of the sample with the lowest content of PANI (PT1100), the peak at ca. 430–440 nm also disappeared. Describing the absorption spectra of samples with high content of polyaniline (PT101, PT501, and PT1001), they were similar to pure PANI, indicating the complete coverage of titania surface.

**Figure 3:**
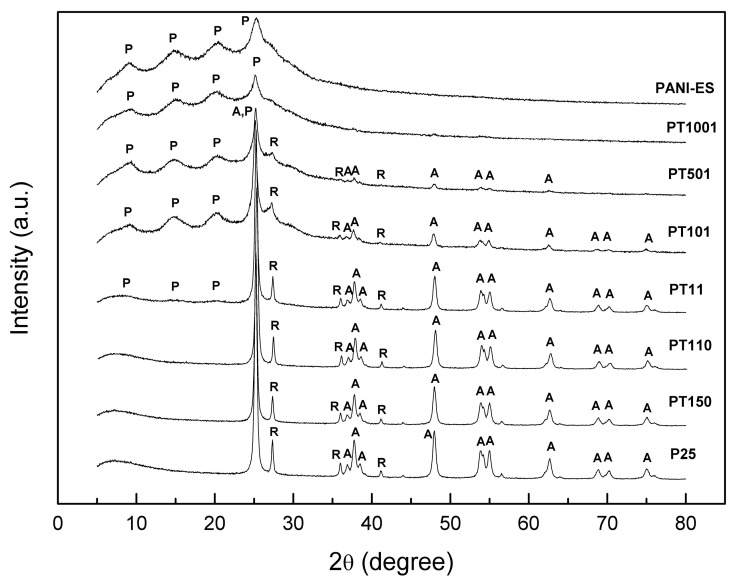
XRD diffractograms of PANI/P25 samples in relation to PANI-ES and P25. A—anatase, R—rutile, and P—polyaniline (emeraldine salt) phases.

The XRD results of the samples considered are shown in Figure 3. XRD analysis of the as-synthesized PANI showed three broad peaks at 9.1°, 14.8°, and 25.3° (Figure 3). This suggests an ES form of polyaniline, and the results were consistent with previous literature reports [50,51,52]. The broad shape of the peaks indicates the predominantly amorphous character of the resulting PANI [44]. For polyaniline-modified TiO_2_ samples, with the exception of samples PT11, PT101, PT501, and PT1001, no clear signals from PANI were observed. This can be justified by the fact that the polyaniline content (in the presence of titania) was below the detection threshold of the diffractometer. The XRD patterns for TiO_2_ were characteristic of P25 and consisted of anatase and rutile peaks (Figure 1). For bare P25, the anatase and rutile phase contents were 85.8% and 14.2%, respectively. The crystallite size of P25 determined from the Scherrer equation (k = 0.891) was 21.5 nm. No significant changes in crystallinity, phase composition, and crystallite size were reported for the polyaniline-modified samples. Only for samples PT101 and PT501 was the accurate determination not possible due to the large amount of PANI that interferes with titania diffractogram. In the case of sample PT1001, no titania peaks were observed. TiO_2_ content was too low, and polyaniline shielded the titania surface.

**Figure 4:**
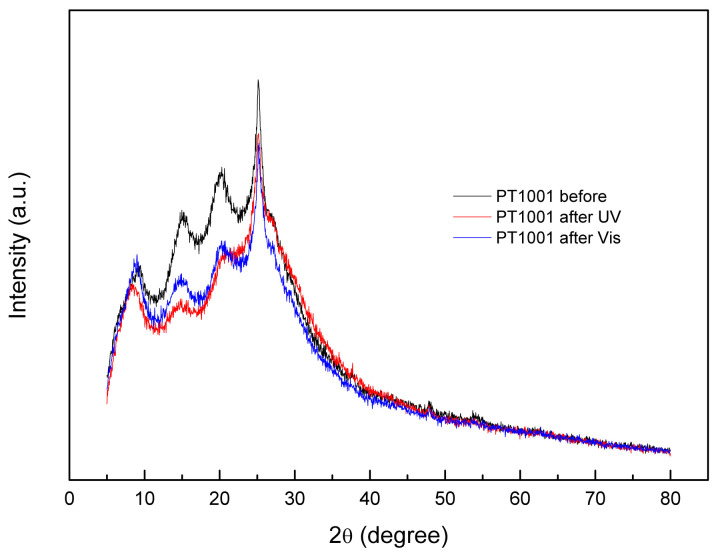
XRD diffractograms of PT1001 sample: before the photocatalytic experiment, after UV, and after visible light-induced experiments.

Through diffractometric studies, the stability of the polyaniline–titania sample (PT1001) was examined. The analysis involved the sample before the photocatalytic experiment, the sample recovered after the UV test, and the sample recovered after the Vis test (Figure 4). The PANI-originated peaks are visible in both recovered samples. A slight decrease in the intensity of these peaks was observed: higher for the sample recovered after the UV photocatalytic experiment and lower for the PT1001 sample recovered after the Vis test. This would indicate a small loss of polyaniline content.

### 2.2. The UV-Induced Photocatalytic Activity of PANI/TiO_2_ Nanocomposites

**Figure 5:**
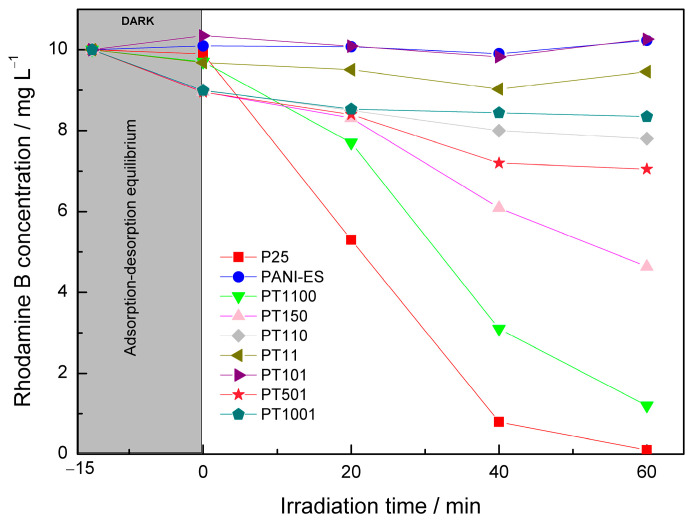
UV-induced photodegradation of RhB in the presence of obtained PANI/TiO_2_ photocatalysts.

**Figure 6:**
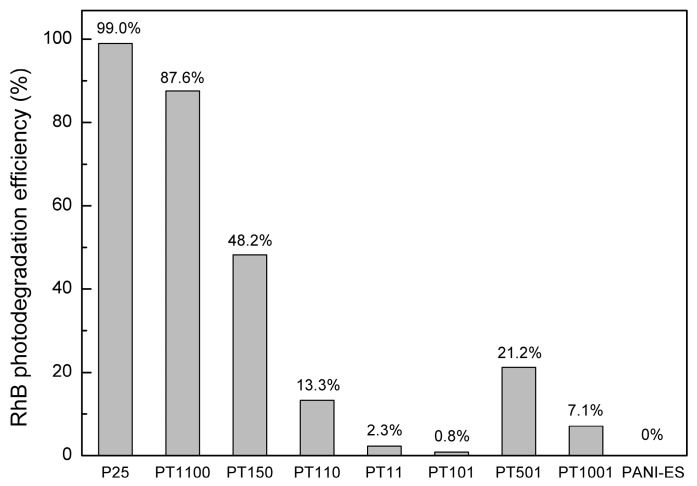
Calculated RhB photodegradation efficiencies in the UV system for considered PANI-based materials.

The photocatalytic properties of the prepared titania-polyaniline heterostructures were tested under both UV and visible light. In the UV system, rhodamine B (RhB) was selected as a model compound. Figure 5 shows the time course of photocatalytic reactions in the presence of the prepared heterostructures in the UV system. The corresponding photodegradation efficiencies are presented in Figure 6. The activities were compared with pure P25 and synthesized bare polyaniline in the ES form (PANI-ES). The most efficient photocatalyst in this system was pure P25. Almost all of the amount of RhB was degraded within an hour. On the contrary, PANI-ES had no UV-induced photocatalytic activity. Figure 6 clearly shows the negative impact of PANI content on photodegradation efficiencies (PEs) of PANI/TiO_2_ materials. PEs of 87.6% and 48.2% were reported for PT1100 and PT150 samples, respectively. In the case of samples with higher content of PANI, PEs in the range of 0.8 to 21.2% were observed.

The superior UV-induced photocatalytic properties of pure TiO_2_(P25) are well known. Titania directly absorbs UV photons, producing electron-hole pairs that move to the surface and react with water and oxygen to yield reactive oxygen species (ROS). Organic molecules are then attacked and oxidized by ROS. Simultaneously, the holes from the valence band of titania can directly oxidize organic compounds [4]. However, PANI can coat the TiO_2_ surface, shielding the transmission of UV light, which blocks contact between the titania surface and organic molecules, causing a decrease in photocatalytic activity. Therefore, it was not possible to find the optimum PANI content for photocatalytic efficiency. No synergistic effect between TiO_2_ and PANI for the UV system exists. There is no consensus in the literature on this topic. Some papers report the positive effect of both components of PANI/TiO_2_ composites and the formation of resulting heterojunctions, which inhibits the charge–carrier recombination [53,54,55,56,57,58]. However, the concept of the role of PANI in the passivation of photocatalytic activity in the UV system was also reported [33,59]. Gu et al. prepared PANI/TiO_2_ materials using polyaniline nanofibers as a template for coupling with titania particles [33]. These photocatalysts, compared to a sample containing PANI nanosheets formed on a TiO_2_ surface, had higher UV-induced photoactivity, but it did not exceed the activity for pure titania. In another work, Sambaza et al. used TiO_2_ with a specific morphology (nanorods), which was used to synthesize PANI/TiO_2_ by a standard oxidative polymerization method [44]. They reported some improvement in the UV-induced photodegradation efficiency for the PANI/TiO_2_ sample compared to pure titania (ca. 19%). Thus, the role of the morphological form of titania and polyaniline interacting at the interface between these components may be important to eliminate the passivation effect. However, the question still remains as to whether effective heterojunction is possible in the UV system.

### 2.3. The Vis-Induced Photocatalytic Activity of PANI/TiO_2_ Nanocomposites

**Figure 7:**
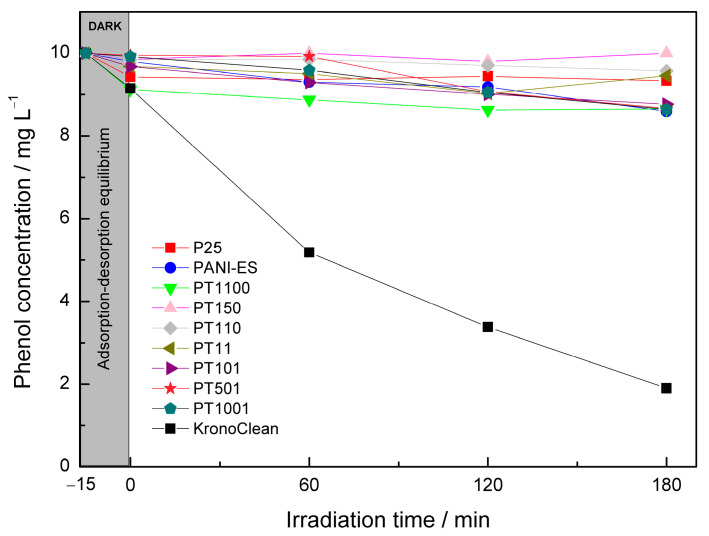
Vis-induced photodegradation of phenol in the presence of obtained PANI/TiO_2_ photocatalysts.

**Figure 8:**
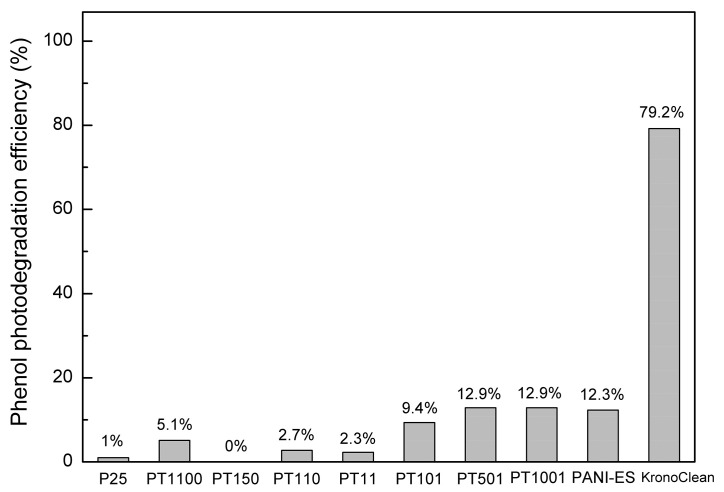
Calculated phenol photodegradation efficiencies in the Vis system for considered PANI-based materials.

The photocatalytic activity of the prepared titania–polyaniline materials was also tested under visible light (4000 K-LED source). In contrast to the previous system, phenol was used as a model organic compound. As mentioned in the introduction, it is not recommended to use dyes in the Vis-system. The photocatalytic activity of the prepared samples in this reaction configuration is shown in Figure 7. Carbon-modified KronoClean titania in the anatase form with visible light activity was used as a reference material. For this sample, 79.2% of phenol was degraded after 180 min of visible light irradiation, justifying the use of an experimental setup based on the selected light source. Unlike the UV system, pure PANI-ES has activity in visible light that is at a low level compared to KronoClean—only 12.3% of PANI-ES degraded. Figure 8 shows the calculated PEs for all samples tested. It can clearly be seen that the higher content of PANI-ES was beneficial for the better visible light activity of PANI/TiO_2_ samples. PEs for PT501 and PT1001 samples were 12.9%, and were close to the efficiency level for pure PANI-ES. It is difficult to conclude a clear improvement in PE here since the difference in the PEs of these two samples is at the level of measurement error. For the sample PT101, the reaction efficiency decreased to 9.4%. In the case of samples with lower PANI content, PEs were observed in the range of 0 to 5.1%. Phenol photodegradation efficiency for pure P25 titania was 1%. Regarding the slightly higher activity of the PT1100 sample (5.1%) compared to the other samples (PT150, PT110, and PT11), due to the low-efficiency value and measurement error, it is difficult to conclude here that there was a significant improvement in activity compared to them and pure P25.

PANI-ES exhibits p-type semiconducting and efficient holes transporting properties [26,60]. Consequently, the photocatalytic properties of this material can be expected, especially in the visible light range. In the present study, PANI-ES showed low activity under Vis, which was significantly higher than that of pure TiO_2_. A similar result was found in the literature [61]. The photoactivity of PANI alone was also reported to be equal to bare P25 [59]. However, in both cases, the exact form of PANI was not determined. Overall, the activity of PANI alone has been investigated in a small number of research studies on PANI/TiO_2_, and this should be the standard to perform a reliable assessment of the photocatalytic properties of the resulting PANI/TiO_2_ materials to determine a real synergistic effect between polyaniline and titania.

Under Vis irradiation, in the PANI/TiO_2_ composites, PANI should act as a photosensitizer. Visible light-induced transitions π→polaron, polaron→π* in the PANI molecules occur. Subsequently, the excited-state electrons from the PANI molecules can be injected into the conduction band of TiO_2_ and then react with oxygen to finally form ROS [58]. Research reports can be found that confirm this mechanism and synergistic effect of polyaniline and titania [32,37,39,59,62]. Only reports using substances other than dyes as model compounds were included in the discussion. The photocatalytic activity of PANI/TiO_2_ samples obtained in this study is related to the PANI-ES content but not to the synergistic effect of the two components. The more PANI-ES in the composite, the higher photocatalytic activity, which for the most active sample is comparable to that of PANI-ES alone. Compared with the literature, Acosta-Alejandro et al. reported a successful synergistic effect of PANI and TiO_2_ in the visible light system using phenol as a model compound [39]. This positive effect was made possible by the formation of mesoporous SiO_2_/TiO_2_ core/shell structure followed by impregnation with PANI-ES. PANI/TiO_2_ sample (1:80) had the highest activity (ca. 36% of PE). In this case, the higher amount of PANI was detrimental to the activity. However, samples based on non-porous material had very low photocatalytic activity, similar to that described in this study. Another successful approach was performed by Liao et al. [32]. They used core-shell mesoporous TiO_2_ microspheres to prepare PANI/TiO_2_ photocatalysts. More PANI molecules were able to undergo chemisorption on the mesoporous surface of titania due to its large specific surface area. Ca. 40% of 4-chlorophenol was degraded after 3 h for a sample containing 6% of PANI. Thus, the mentioned successful attempts to obtain PANI/TiO_2_ materials with good photocatalytic properties in the Vis system (for colorless model organic compounds) are related to the application of mesoporous surface and/or high specific surface area of base titania material. The results obtained in this study confirm that the use of standard titanium dioxide (e.g., P25) without surface modification and with an average surface area does not favorably affect the interaction between polyaniline and TiO_2_, hindering the formation of a synergistic effect.

## 3. Materials and Methods

### 3.1. Materials

Commercially available TiO_2_ (P25; Aeroxide^®^ P25, Evonik, Essen, Germany), consisting of anatase and rutile, was used without surface pretreatment for the modification with polyaniline. KRONOClean^®^ 7000, a commercially modified anatase (KronoClean; Kronos Worldwide Inc., Dallas, TX, USA), was used as a reference for visible light-induced photocatalytic experiments. Aniline (98%, Sigma-Aldrich, Darmstadt, Germany), ammonium persulfate (98%, Sigma-Aldrich, Darmstadt, Germany), and hydrochloric acid (36.5%, POCh, Gliwice, Poland) were used as starting materials for the polymerization procedure. Phenol (99%, Sigma-Aldrich, Darmstadt, Germany) and rhodamine B (RhB; 99%, Sigma-Aldrich, Darmstadt, Germany) were selected as model organic compounds.

### 3.2. Preparation Procedure of PANI and PANI/TiO_2_(P25) Photocatalysts

PANI/TiO_2_(P25) samples were synthesized by the in situ oxidative polymerization of aniline monomer using ammonium persulfate as a redox initiator. Solution A was prepared by adding the specified amount of P25 to 90 mL of 1 M HCl solution in a flat-bottomed flask, and the resulting suspension was subjected to ultrasonication for 30 min. Solution B was prepared by dissolving 0.6524 g of APS in 30 mL of 1 M HCl solution and placed in a dropping funnel. Solution A was placed in an ice bath, and 0.233 g of aniline was slowly added via vigorous stirring provided by a magnetic stirrer. Solution B was added dropwise (1 drop/s) into solution A. Continuous stirring was carried out for another 24 h at room temperature. The precipitate of the PANI/TiO_2_(P25) composite was filtered through a Gooch funnel equipped with a sintered glass disc, washed extensively first with distilled water and then ethanol, and dried in an air dryer at 80 °C for at least 24 h until constant weight was reached. The following molar ratios between aniline and P25 were included in this study: 100:1, 50:1, 10:1, 1:1, 1:10, 1:50, and 1:100. The corresponding names of samples are: PT1001, PT501, PT101, PT11, PT110, PT150, and PT1100. The same preparation procedure was also used for the synthesis of bare PANI in the absence of TiO_2_.

### 3.3. Photocatalytic Activity

The photocatalytic activity of the prepared samples was evaluated in two systems: under UV and visible light (Vis). Model organic compounds were selected depending on the system: RhB for UV and phenol for Vis. The initial concentration of model compounds was 10 mg/L. In the first stage, a suspension of the selected samples (1 g/L) in a system containing the aforementioned compounds was prepared. At first, the prepared suspension was placed in the dark and stirred for 15 min to establish adsorption–desorption equilibrium. Then, the corresponding light source (UV or Vis) was turned on. In the case of the UV system, an LED lamp emitting ultraviolet radiation at a wavelength of 395 nm (Bridgelux, Fremont, CA, USA) with a power of 50 W was used. The light source was placed at the top relative to the sample and equipped with an active cooling system, which consisted of an aluminum radiator and a fan. The presence of these elements was intended to dissipate the generated heat and thus ensure a stable temperature during the test of photocatalytic activity. For the Vis system, an LED light source in the form of a strip consisting of 60 LEDs (type SMD5630, Samsung, Seoul, Republic of Korea) with a total power of 18 W and a color temperature of 4000 K was used. The position of the exposed sample was central. Cooling was provided by a fan. In both systems, the temperatures of the irradiated photocatalyst suspension did not exceed 30 °C. During the experiment, 3 mL liquid samples were collected after 0, 20, 40, and 60 min (UV system) and 0, 60, 120, and 180 min (Vis system). All liquid samples were filtered through a 20 μm syringe filter (Chromafil^®^ PET 20/25, Macherey-Nagel, Düren, Germany). The concentration of phenol was determined by the colorimetric method that involves diazotization of 4-nitroaniline and subsequent coupling of the diazonium chloride with phenol. The colored diazo compound formed was determined by measuring the absorbance at 477 nm using a UV-Vis spectrophotometer (V-750, Jasco, Tokyo, Japan). Rhodamine B determination based on direct absorbance measurement at 541 nm. Photodegradation efficiency (PE) was calculated using Equation (1):

(1) $$PE\;\left[\%\right]=\left(1-\frac{C_{t}}{C_{0}}\right)\cdot 100\%$$

where *C*_0_ (mg/L) is the concentration of the model compound at time 0 min (switching on the lamp), and *C_t_* (mg/L) is the concentration at time *t* when the light source is switched off.

### 3.4. Characterization of Prepared Materials

UV-Vis diffuse reflectance measurements were conducted at room temperature on finely ground samples. Spectra were recorded in the range from 200 to 800 nm with a Jasco V-770 UV-Vis-NIR spectrophotometer (Jasco, Tokyo, Japan) equipped with ISN-923 integrating sphere using Spectralon as a reference. The measured reflectance spectra were subsequently converted to Kubelka–Munk (K-M) absorption factors to evaluate the absorption spectra of the samples.

X-ray diffraction (XRD) analyses were performed by a Rigaku SmartLab SE X-ray diffractometer (Rigaku, Tokyo, Japan) operating in the reflection mode with Cu-Kα radiation at 40 kV and 30 mA and equipped with a Hypix-400 detector (Rigaku, Tokyo, Japan). XRD data were collected over the 2Ɵ range of 5–80° at a rate of 4° min^–1^ and with a 0.04° step resolution. Phase composition and crystallite sizes were determined through SmartLab Studio II software (Rigaku, Tokyo, Japan).

## 4. Conclusions

Given the diverse and inconclusive literature reports on the photocatalytic activity of PANI/TiO_2_ heterostructures and the synergistic effect between the components, a series of PANI/TiO_2_ photocatalysts with different polyaniline contents were prepared by in situ chemical oxidative polymerization of aniline with APS in the presence of TiO_2_(P25). The resulting polyaniline was identified as an emeraldine salt. The photocatalytic activity of the prepared materials was evaluated in two reaction systems induced by UV and visible light. In the UV system, none of the prepared samples had activity better than P25. It has been observed that the presence of PANI-ES adversely affected the photocatalytic properties since the PANI-ES layer covering the titania surface can shield the UV light transmission by blocking the contact between the TiO_2_ surface and organic molecules. The design of polyaniline and titania nanostructures with specific morphological forms can improve the interaction between these components at the interface and, consequently, eliminate the photocatalytic activity passivation effect. When visible light was used, no synergy was observed between PANI-ES and TiO_2_. Phenol was used as a model compound instead of dyes, which have been frequently used in the literature. The photocatalytic properties of the most active composite were similar to those of pure polyaniline. Referring to literature reports, it can be concluded that the application of base titania material with a mesoporous surface and/or a high specific surface area can allow the obtaining of materials with real visible light activity. In conclusion, in order to obtain PANI/TiO_2_ composite materials active in UV and/or visible light, it would be necessary to take into account the morphological and surface properties of both components to achieve the synergistic effect between the components.

## Data Availability

The data presented in this study are available from the corresponding author upon reasonable request.
